# Factors affecting consumers’ purchase intention for agriculture products omni-channel

**DOI:** 10.3389/fpsyg.2022.948982

**Published:** 2023-01-03

**Authors:** Yan Liu, Shuyin Zheng

**Affiliations:** ^1^College of Economics and Management, Huanghuai University, Zhumadian, China; ^2^School of Management, Shanghai University, Shanghai, China

**Keywords:** COVID-19 pandemic, purchase intention, omni-channel, structural equation model, value-based adoption model

## Abstract

Agricultural retailers face serious challenge of losing consumers due to channel integration, it has become essential to provide an expected consistent and seamless omni-channel shopping experience in an omni-channel environment. Although previous literature has begun to focus on the consumer perspective of the omni-channel experience, little attention has been paid to the process of change from a single retail environment to omni-channel. By combining the research frameworks of unified theory of acceptance and use of technology (UTAUT) and the value-based adoption model (VAM), this study aims to identify the drivers of omni-channel consumer purchase intention in agriculture products retail. This article conducts a questionnaire survey on 620 samples in China. The results show that single-channel shopping cost, reference groups, positive online reviews, and single-channel perceived risk have a significant positive impact on the omni-channel purchase intention of agricultural products, and perceived value plays a mediating role. Moreover, contextual factors partially have a negative moderating effect. When consumers purchase agricultural products, the more suitable the online shopping environment is, the weaker the influence of single-channel shopping costs, reference groups and positive online reviews on consumers’ omni-channel purchase intention. These findings build on the existing literature on the omni-channel retail consumer experience and provide insights for fresh produce retailers to implement and evaluate an omni-channel integration strategy for agriculture products. The findings may shed lights on how to promote the healthy development of the omni-channel sales model of agricultural products.

## Introduction

The spread of COVID-19 drives a huge surge in demand for agricultural products, however, the challenges surrounding the production, service, logistics and labor availability of agricultural products have posed a constant threat (De and Singh, 2021). As a result, the key adjustment for marketers is to move from single-channel to omni-channel collaboration in managing consumer relationships, in other words, using goods to attract people (Huré et al., 2017). Chinese retailers are attempting to innovate their produce distribution models to digitally engage consumers and create a unified and seamless experience across intermingled touch points (Morgenstern, 2021), such as live-streaming vegetable sales, contactless deliveries, and smart food baskets.^1^ Clearly this demand has been amplified by the enhanced ability of consumers to choose their channels. Digital News Asia (2019) data shows that 90% of Asian consumers prefer a combination of channels to purchase goods (mobile, app, in-store or desktop). By the end of 2021, the scale of fresh food e-commerce transactions will reach 68.9 billion dollars, a year-on-year increase of 27.92%. The penetration rate of the fresh food e-commerce industry reached 7.91% in 2021.^2^ This new omni-channel environment is blurring the boundaries between offline and online retailing, with each channel taking on a networked distribution to cater consumers’ purchasing needs across time and geography (Zhang et al., 2021). Meeting these expectations can be challenging for retailers, however, if successfully achieved, retailers will improve sales growth and improve customer retention (Fang et al., 2021).

In the traditional agricultural distribution system, farmers are connected to markets through the Farmers-Origin Wholesale Market—Retail Terminal (Ming et al., 2021). However, in China, the long distribution chain and the high cost of transportation and storage often result in negative phenomena—contradictions between supply and demand, slow transmission of information in the supply chain and narrowing of suppliers’ access to goods (Liang et al., 2022). With the development of e-commerce and information technology, from the earliest days when there was only a single channel, it evolved to dual channels, multiple channels and eventually to the current common omni-channel. The omni-channel agricultural supply chain that this study concerned usually consists of a single farmer cooperative and one online retailer offering three shopping options, namely pick-your-own, online purchase and home delivery (Hu and Xu, 2019).

In omni-channel shopping, consumers not only get the same quality of products as in fresh produce shops, but also the convenience of e-commerce (Zhang et al., 2020). Also, integrating online-offline channels offers an innovative way of thinking to solve the “last mile” problem of fresh produce (Janjevic et al., 2021; Wang et al., 2022). However, the plans to connect, collaborate and synchronize the various channels are large and complex for retailers (Abrudan et al., 2020), so it is necessary to measure consumer value to assess the relevance of these efforts (Huré et al., 2017). It will promote better planning by retailers and increase the knowledge of consumers’ willingness to buy agricultural products.

Although omni-channel retailing has generated a lot of interest among marketing researchers, it needs to be explored further in the fresh agri-produce sector. Existing research has focused on pricing and service level decisions in the fresh produce supply chain (Yu and Xiao, 2017; Jin et al., 2018), logistics distribution models (Ailawadi and Farris, 2017; Wollenburg et al., 2018) and digital strategic governance (Saghiri and Mirzabeiki, 2021; Zhang et al., 2021). For example, Jin et al. (2018) explored omni-channel supply chain pricing and order fulfillment on buy–online–pick-up-in-store mode. This illustrates the importance of coordination and optimization of the omni-channel supply chain. In addition, a small number of empirical studies in other areas have begun to recognize shopping value of omni-channel (Murfield et al., 2017; Pallant et al., 2020). Huré et al. (2017) identifies three dimensions for accurately measuring omni-channel shopping value: utilitarian, hedonic and social dimensions. Researchers have also demonstrated that personal innovativeness, effort expectancy, and performance expectancy influence omni-channel consumers’ behavior (Juaneda-Ayensa et al., 2016). But they rarely focus on the process of change from a single retail environment to an omni-channel. What influences consumer behavior? And how retailers are guiding consumers through the transition to an omni-channel environment and how they can leverage various channels to maximize consumer value?

Unlike other industries, fresh produce has a short life cycle, a high wastage rate and market demand fluctuates with the freshness of the product (Zhou et al., 2020; Ming et al., 2021); therefore, providing easier and more comprehensive perceived value is key to innovation in the agricultural supply chain. Moreover, in an omni-channel environment, the characteristics and attributes of touch points vary and consumer perceptions of them are relative (Stein and Ramaseshan, 2016; Huré et al., 2017). In general, based on the perspective of single touch points and other social impacts, we attempt to revisit the focus of omni-channel value measurement. Specifically, this study aims to answer the following three research questions: *(1) Do single-channel shopping costs, reference groups, positive online reviews and single-channel perceived risk affect consumers’ perceived value and willingness to purchase agricultural products through omni-channel? (2) Do contextual factors moderate the relationship between perceived value and purchase intention to purchase agricultural products omni-channel? (3) What are the prerequisites for purchasing agricultural products omni-channel?*

## Literature review and research model

### Omni-channel retailing of agricultural products

Understanding the significance of omni-channel for agricultural products is a pre-condition for capturing the value of omni-channel shopping among consumers. Rigby (2011) first introduced omni-channel concept in Harvard Business Review, which states that retailers integrate all available channels (e.g., stores, websites, social media, TV, etc.) to communicate, transact and co-create value with consumers. Compared with single channel or multi-channel, the unique aspects of omni-channel are: multiple interactions across channels and touch points; focus on the interaction between channels and brands; and meet consumers’ expectations for a seamless shopping experience (Huré et al., 2017; Jin et al., 2018). Verhoef et al. (2015) defined omni-channel management as “synergistic management of the numerous available channels and customer touchpoints, in such a wat that the customer experience across channels and the performance over channels is optimized.” Consumers can switch retail channels at multiple stages of the purchase journey based on their personal preferences, and ultimately complete shopping behaviors such as information search, product purchase, shipping, and returns (Asmare and Zewdie, 2022). Therefore, retailers need to develop omni-channel strategies in response to consumers’ changing purchase methods (e.g., in-store or online purchases) and preferences (e.g., home delivery or pick-up in store) (Murfield et al., 2017).

In the field of agricultural products, considering that channel demand is influenced by product freshness and price, researchers have tried to solve difficult supply chain problems (e.g., risk aversion and decision making) (Yu et al., 2018; Yan et al., 2020; Feng et al., 2021). But these studies side-by-side demonstrate the value of omni-channel, for example, that cross-channel integration can contribute to improved cost effectiveness (Hu and Xu, 2019). The above research provides a framework for further research in the area of agricultural products omni-channel retailing, but their usefulness in guiding practice is limited. To fill this gap, we theoretically investigate the impact of single-channel value of fresh produce and other social influences on the shopping value of consumers. And it provides insights in promoting the development of marketing strategies for agricultural products.

### Unified theory of acceptance and use of technology and value-based adoption mode

Most current retail research on new technology acceptance the theory of reasoned action (TRA), the theory of planned behavior (TPB), unified theory of acceptance and use of technology (UTAUT) and the technology acceptance model (TAM) to explore consumers’ purchase intentions (Herrero-Crespo et al., 2022; Kim et al., 2022; Mathavan et al., 2022).

Venkatesh and Davis (2000) extended TAM and effectively explained perceived usefulness and technology use intentions through social influence and cognitive processes. Social influence is defined as the degree to which an individual perceives that important others believe he or she should use the new system. Venkatesh et al. (2003) further constructed UTAUT, including four key elements, performance expectations, effort expectations, social impact, and convenience. UTAUT has been shown to predict technology acceptance among automotive retail users in an omni-channel context (Kim et al., 2022). One manifestation of implementing an omni-channel strategy is replacing some big stores with online stores. It can reduce operating costs and facilitate the integration of online and offline channels (Kazancoglu and Aydin, 2018). But some consumers are still skeptical of such a model, which implies that contextual factors may influence consumers’ decision-making processes in an omni-channel context (Daugherty et al., 2019). In this regard, this study focuses on the social impact of UTAUT, exploring the role of reference groups, positive online reviews.

**FIGURE 1:**
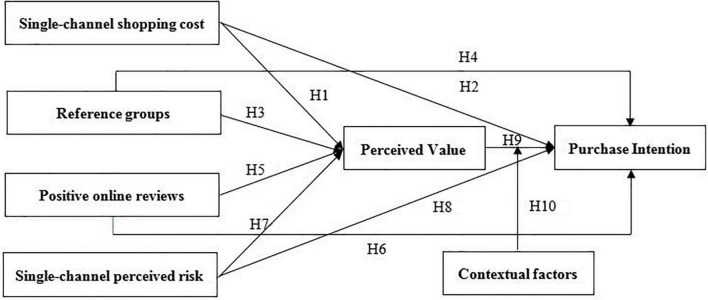
Research framework and hypotheses.

Kim et al. (2007) argued that TAM is considered for adoption from the perspective of technology users, while consumers differ from technology users in terms of bearing costs and risks, and therefore proposed the value-based adoption model (VAM). And he analyzed consumers’ adoption of new technologies and their purchase behavior in the e-commerce technology environment from the perspective of value maximization. VAM has been found to be effective in terms of intention to use (Vishwakarma et al., 2020). Overall, considering the special attributes of agricultural products, channel characteristics, channel transfer costs, and changes in perceived value, this study uses a combined framework consisting of UTAUT and VAM, which is more suitable in predicting consumer purchase intention. To be specific, the purpose of this study is to investigate the effects of single-channel shopping costs, single-channel perceived risk, reference groups, and positive online reviews on perceived value and omni-channel purchase intentions. The conceptual model of this paper is shown in Figure 1.

## Hypothesis development

### Single-channel shopping cost

As channel choice opportunities increase, consumers begin to consider the opportunity costs associated with shopping activities, such as product prices, search costs, switching costs, and transportation costs (Lim et al., 2011; Chang et al., 2017; Singh and Jang, 2022). Price is the currency that consumers pay to obtain a product or service (Wood and Scheer, 1996). Many scholars have studied the impact of price on the sales ability of enterprises. Forman et al. (2008) analyzed Amazon’s data and found that online retail prices can significantly affect retailers’ sales. Many previous studies have shown that online channels can effectively reduce consumers’ purchasing costs. Peterson and Merino (2003) pointed out that search cost refers to the time and physical effort that consumers spend in collecting product information during the shopping process. The research of Jepsen (2007) shows that consumers can search for information about purchasing products through online channels, which can break through the limitations of time and space and reduce the search cost. The dissemination of product quality information to consumers breaks through the limitations of time and space, which reduces the search cost of consumers and helps to increase their willingness to buy (Heng et al., 2018). Zheng et al. (2020) pointed out that competitive prices are a key factor for consumers to choose to buy agricultural products online. Zatz et al. (2021) compared consumers’ omni-channel purchase behavior and found that consumers are more likely to compare prices online, save time and convenience, improve consumers’ perceived value, and increase their willingness to buy. When purchasing agricultural products omni-channel, consumers can easily obtain all product-related information, effectively saving time and transportation costs. Consumers can also quickly obtain product information from successfully traded orders, further reducing search and switching costs in the purchasing process (Pozzi, 2012; Richards et al., 2017). Therefore, it is hypothesized that:

H1: Single-channel shopping costs have a significant positive effect on the perceived value of purchasing agricultural products omni-channel.

H2: Single-channel shopping costs have a significant positive effect on the purchase intention to purchase agricultural products omni-channel.

### Reference groups

A reference group is a person or group of people who significantly influences an individual’s behavior. This concept is widely used in marketing strategies by marketing practitioners to influence consumers’ product and brand decisions (Bearden and Etzel, 1982; Bearden et al., 1989). (Amaldoss and Jain, 2005a,b, 2015) demonstrated that reference group effects can affect the retail price of firms and the branding decisions of consumers. Studies have shown that reference groups can directly affect individual behavior (Dimanche and Havitz, 1995; Welsch and Kühling, 2009). Escalas and Bettman (2005) pointed out that consistency within reference groups will have a significant positive impact on consumers’ brand perception. Sun et al. (2022) demonstrated that the strength of reference-group effects affects consumers’ product decisions. Thus, the firm will adopt a different introduction strategy for upgraded products. Therefore, it is hypothesized that:

H3: Reference groups have a significant positive effect on the perceived value of purchasing agricultural products omni-channel.

H4: Reference groups have a significant positive effect on the purchase intention of purchase agricultural products omni-channel.

### Positive online review

Online reviews are a kind of interpersonal communication. Consumers transmit information related to products or sellers’ services through the Internet based on their own product purchase and use experiences (Hennig-Thurau et al., 2010). Some scholars have found that positive reviews can affect consumers’ perceived value. Online reviews provide consumers with more information, which is helpful for them to identify the quality of products, judge the ability of products to meet their needs, and eliminate their concerns (Kwark et al., 2014). Online reviews affect consumers’ perceived value, and choosing online review management platform services has become a consensus among B2C companies (König et al., 2022). Other studies have shown that positive reviews further influence consumer purchase intentions. Bao and Chang (2014) found that the effective use of online reviews by companies can increase sales. Online reviews will bring value-added effects to consumers, improve the accuracy of their judgments on product quality, and increase consumers’ purchasing intentions (Fan et al., 2020). In the era of mobile internet, nearly 95% of consumers will read online reviews before or during their purchasing decisions (Tom et al., 2020). Therefore, it is hypothesized that:

H5: A positive online review has a significant positive effect on the perceived value of purchasing agricultural products omni-channel.

H6: A positive online review has a significant positive effect on the purchase intention of agricultural products omni-channel.

### Single-channel perceived risk

Perceived risk is the consumer’s expectation of loss when making a purchasing decision (Stone and Grønhaug, 1993). Corbitt et al. (2003) believed that perceived risk affects consumer trust, which in turn affects purchase intention. Online channels cannot directly contact products, and virtual product information displays may be perceived as accurate enough to make suitable judgments (Park et al., 2005). Bennett and Harrell (1975) suggest that lower perceived risk may be related to higher purchase intention. Vijayasarathy and Jones (2000) found that perceived risk plays an important role in the process of making purchase decisions. Ueland et al. (2012) proposed that consumers’ willingness to purchase products depends on perceived risks and perceived benefits. Therefore, the following hypothesis was developed:

H7: Single-channel perceived risk has a significant positive effect on the perceived value of purchasing agricultural products omni-channel.

H8: Single-channel perceived risk has a significant positive effect on the purchase intention to purchase agricultural products omni-channel.

### Perceived value and purchase intention

The perceived value constructed by Kim et al. (2007) includes the price paid to purchase the product, the usefulness of the product, and the hedonicity of the product. The selling price of a product directly affects perceived value (Zeithaml, 1988; Dodds William et al., 1991). Early research on perceived value focused on perceived quality and price (Chang and Wildt, 1994; Grewal et al., 1998).

Individuals evaluate the consequences of their actions based on perceived usefulness and choose actions based on the desirability of usefulness. The construction of usefulness is similar to the marketing concept of product quality, which is defined as the customer’s cognitive assessment of product excellence or superiority (Voss et al., 1998). Consumers believe that purchasing agricultural products omni-channel is more conducive to identifying product attributes, which are products with functions that satisfy them. Steenkamp (1990) defines product quality as fit for consumption, that is, the usefulness of a product in meeting consumer needs.

With the upgrade of consumption, consumers purchase products to obtain the functional value and hedonic value of the product (Babin et al., 1994). Davis et al. (1989) pointed out that hedonic value refers to the degree of pleasure brought to consumers by the activities of purchasing and using a product, excluding any expected functional value. Hedonic value represents intrinsic, emotional value. In addition to product usefulness, hedonic value is also an important component of perceived value (Davis et al., 1992). Hedonic value has a positive effect on perceived value (Petrick, 2002).

Neal’s (1999) study shows that perceived value will bring customer loyalty, generate purchase behavior, and further improve customer loyalty. Customer perceived value has a direct impact on purchase intention (Cronin et al., 2000; Sweeney and Soutar, 2001). Therefore, it is hypothesized that:

H9: Perceived value has a significant positive effect on the purchase intention of purchasing agricultural products omni-channel.

### Moderating effects of contextual factors

Contextual factors are the key factors affecting consumers’ purchase intention (Dhar, 1992). Contextual factors can influence consumers’ purchasing decisions to deviate from stable preferences (Prelec et al., 1997). Wu and Wang (2005) pointed out that in the process of transforming attitude into behavior, it will be affected by contextual factors. Contextual factors such as the price, availability, and convenience of purchasing channels of agricultural products can affect consumers’ attitudes and then determine their purchase intentions (Zepeda and Deal, 2010). Zhu et al. (2013) found that contextual factors such as the convenience of purchase significantly moderate the influence between consumers’ purchase intention and purchase behavior. Therefore, it is hypothesized that:

H10: Contextual factors will positively moderate the relationship between perceived value and purchase intention of purchase agricultural products omni-channel.

## Research methodology

### Instrument

The design of the variable scales in the questionnaires in this paper draws on published research papers and is adapted from mature scales combined with the results of market interviews. The measurement items for single-channel shopping cost were adapted from Wood and Scheer (1996) and Jepsen (2007). reference groups was measured using four items adapted from Bearden and Etzel (1982) and Escalas and Bettman (2005). Positive online reviews were measured using five items adapted from Hennig-Thurau et al. (2010) and Fan et al. (2020). Single-channel perceived risk was measured using five items adapted from Stone and Grønhaug (1993) and Corbitt et al. (2003). The measurement of perceived value draws on the studies of, Davis et al. (1992); Voss et al. (1998), and Neal (1999). The measurement of purchase intention draws on the research of Cronin et al. (2000) and is designed from three aspects: willingness to shop omni-channel, possible omni-channel shopping in the future, and recommending others to shop omni-channel. The measurement of online contextual factors draws on the studies of Dhar (1992) and Zepeda and Deal (2010) and is designed from four aspects: purchasing convenience, price, availability, and policies and regulations.

As the original items were in English, we conducted a back translation to ensure translation validity. First, a researcher whose native language was Chinese translated the source items from English into Chinese. Next, another researcher independently translated these items back into English. Subsequently, the two researchers compared the two English versions and jointly revised the first Chinese version of the items. Based on their feedback, minor modifications were made to improve the comprehensiveness and user friendliness of the measurement items. To ensure the reliability and validity of the scale, the research team first consulted experts in relevant fields to propose revisions and then conducted a preliminary survey. The scale was revised according to expert opinions and preinvestigation results, and finally, a formal scale was formed. The final survey questionnaire is presented in Appendix. The dimensionality of all variables are unidimensional, and all items were measured on a 7-point Likert scale, which ranged from 1 (not agree at all) to 7 (absolutely agree).

### Data collection and sample

All items were measured on a 7-point Likert scale, which ranged from 1 (not agree at all) to 7 (absolutely agree). To ensure the reliability and validity of the scale, the research team first consulted experts in relevant fields to propose revisions. We conducted a preliminary survey on the questionnaire with volunteers who have channel switch experience in purchasing agricultural products (146 volunteers in total). The scale was revised according to expert opinions and pre-investigation results, and finally, a formal scale was formed. The final survey questionnaire is presented in Appendix A.

A power analysis using R*power 3.6 computer software was conducted to calculate a suitable sample size (Hua et al., 2020). The results indicated that a sample size of 168 would be sufficient using a one-tailed test and a power (1 – β) = 0.95, given α = 0.05. The survey covered 635 respondents who were selected randomly from the consumers of Missfresh in China who were members of the firm’s site or app. The official survey of the data in this article will be conducted from December 2021 to March 2022. After purification of the questionnaire, incomplete answers were excluded or the same answer was selected for all the question items. We selected consumers who had either the experience or intention of purchasing agricultural products. Finally, we received 620 valid questionnaires, accounting for 93.37% of all the returned questionnaires.

The reasons for choosing Missfresh are as follows. Missfresh was established in November 2014 and was invested by Tencent. It is a technology-driven innovative community retail enterprise dedicated to allowing every family to buy with peace of mind and eat with confidence. As of April 2021, Missfresh ranked first in both the number and amount of financing events for fresh food e-commerce projects in China. Missfresh pioneered the “prewarehouse” model, providing “over 4,000 items of products to tens of millions of households in 16 cities, within 30 min of delivery.”

The demographic information of the final sample is summarized in Table 1. The gender distribution of the samples is dominated by female participants (77.26%), male participants (22.74%). The age distribution is mainly between 25 and 35 years old (82.26%). The monthly income of the sample is (unit: $), below 442.2 (19.53%), 442.2∼737 (49.74%), 737∼1,474 (24.48%), 1,474∼2,948 (4.95%). The most sample were highly educated, including undergraduate (83.87%), post-graduate or above (5.00%), junior college (5.64%), high school or below (5.48%). Dominici et al. (2021) point out that a young, well-educated, female consumer with a very good or adequate overall economic condition will be more likely to buy food online. Overall, the sample structure is basically consistent with the characteristics of cross-channel purchaser agricultural products, and the sample has good typicality and representativeness.

**TABLE 1:** Demographics of the survey respondents (*N* = 620).

Demographics	Category	Frequency	%
Gender	Male	141	22.74
	Female	479	77.26
Age	≤25	64	10.32
	26–35	510	82.26
	36–55	32	5.16
	≥56	14	2.26
Education	High school or below	37	5.97
	College student	515	83.06
	Graduate school or above	68	10.97
Income	≤442.2	121	19.53
	442.2∼737	308	49.74
	737∼1,474	152	24.48
	1,474∼2,948	31	5
	≥2,948	8	1.3

## Data analysis and results

In this paper, SPSS 20.0 and AMOS 22.0 software were used to process the data and construct a structural equation model (SEM). First, this paper tests reliability and validity, including reliability, convergent validity and discriminant validity. Next, the paper constructs a SEM to examine the path coefficients between latent variables. Finally, this paper uses SPSS PROCESS v3.3 to test the moderating effect of contextual factors.

### Assessment of construct measurements

This paper implemented the evaluation of measurements based on their internal consistency reliability, convergent validity, and discriminant validity. Cronbach’s α and composite reliability (CR) are adopted to test internal consistency reliability (Fornell and Larcker, 1981; Hua et al., 2020). As shown in Table 2, the Cronbach’s α coefficient of all measurement items is above 0.701∼0.928, indicating a high level of reliability. The Cronbach’s α coefficients of the latent variables of single-channel shopping cost, reference groups, positive online reviews, single-channel perceived risk, perceived value, purchase intention, and contextual factors are 0.851, 0.798, 0.929, 0.928, 0.875, 0.911, and 0.847, respectively. The CR coefficients of all latent variables are greater than 0.80. The results indicate that all scales designed in this paper can reliably measure latent variables. The scales used in this paper are all representative mature scales, which are adopted from the published documents in Authoritative journals. Therefore, they have good content validity. Convergent validity is the extent of the positive relation between a measure and alternative measures of the same construct (Hair et al., 2014). The average variance extracted (AVE) of the variables was greater than 0.500, the factor loading of each observed variable exceeded 0.700, and the *t*-values all reached the significance level of *p* < 0.05 (*t* > 1.960), indicating that each variable had good convergent validity.

**TABLE 2:** Construct reliability and convergent validity.

Variable	Items	Loading	CR	AVE	Mean	SD
Single-channel shopping cost α = 0.851	SSC1	0.855	0.903	0.699	5.467	1.384
	SSC2	0.872				
	SSC3	0.795				
	SSC4	0.820				
Reference groups α = 0.798	RG1	0.866	0.874	0.636	5.096	1.417
	RG2	0.867				
	RG3	0.701				
	RG4	0.741				
Positive online reviews α = 0.929	POR1	0.866	0.947	0.781	5.430	1.278
	POR2	0.870				
	POR3	0.895				
	POR4	0.902				
	POR5	0.884				
Single-channel perceived risk α = 0.928	SCP1	0.901	0.946	0.778	4.858	1.586
	SCP2	0.913				
	SCP3	0.912				
	SCP4	0.854				
	SCP5	0.826				
Perceived value α = 0.875	PV1	0.796	0.911	0.671	5.160	1.345
	PV2	0.841				
	PV3	0.827				
	PV4	0.849				
	PV5	0.781				
Purchase intention α = 0.911	PI1	0.928	0.944	0.850	5.397	1.127
	PI2	0.928				
	PI3	0.909				
Contextual factors α = 0.847	CF1	0.837	0.900	0.692	5.398	1.217
	CF2	0.763				
	CF3	0.861				
	CF4	0.863				

Discriminant validity refers to the extent to which a construct is truly distinct from others by empirical standards (Hair et al., 2014). This paper tests discriminant validity by comparing the correlation coefficient between the square root of the AVE value of the latent variable and other variables. As shown in Table 3, the square root of the AVE value of each construct’s variance inflation factor (single-channel shopping cost, reference groups, positive online reviews, single-channel perceived risk, perceived value, purchase intention, and contextual factors) is greater than its highest correlation coefficient with other variables, indicating that each latent variable’s discriminant validity was adequate.

**TABLE 3:** Construct discriminant validity.

Variable	Square root of AVE	1	2	3	4	5	6	7
1. SSC	0.836	1						
2. RP	0.797	0.553**	1					
3. POR	0.884	0.477**	0.569**	1				
4. SCP	0.882	0.533**	0.535**	0.399**	1			
5. PV	0.819	0.618**	0.565**	0.507**	0.687**	1		
6. PI	0.922	0.651**	0.554**	0.576**	0.520**	0.724**	1	
7. CF	0.832	0.653**	0.580**	0.547**	0.539**	0.726**	0.818**	1

**P < 0.01. SSC, single-channel shopping cost; RG, reference groups; POR, positive online reviews; SCP, single-channel perceived risk; PV, perceived value; PI, purchase intention. CF, contextual factors.

### Structural equation modeling analysis

#### Data analysis with structural equation model

**FIGURE 2:**
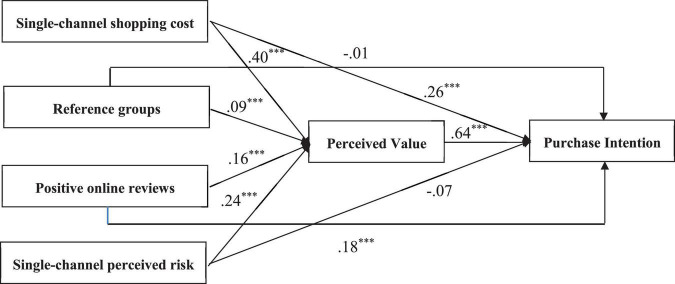
Path coefficient diagram of structural equation model. ***P < 0.001.

AMOS 22.0 software is used to construct a SEM to test the conceptual model and the hypotheses shown in Figure 1. The test results of the hypotheses between single-channel shopping cost, reference groups, positive online reviews, single-channel perceived risk, perceived value, and purchase intention are shown in Table 4. As shown in Table 4, single-channel shopping cost has a positive influence on perceived value (β = 0.4, *p* < 0.001). Thus, hypothesis 1 is supported. In support of hypothesis 2, single-channel shopping cost has a positive influence on purchase intention (β = 0.26, *p* < 0.001). In support of hypothesis 3, reference groups have a positive influence on perceived value (β = 0.09, *p* < 0.05). In support of hypothesis 5, positive online reviews have a positive influence on perceived value (β = 0.16, *p* < 0.001). In support of hypothesis 6, positive online reviews have a positive influence on purchase intention (β = 0.18, *p* < 0.001). In support of hypothesis 7, single-channel perceived risk has a positive influence on perceived value (β = 0.24, *p* < 0.001). In support of hypothesis 9, perceived value has a positive influence on purchase intention (β = 0.64, *p* < 0.001). The overall fitting index of the model basically meets the requirements of the SEM, CMIN/DF = 2.42, less than 3, RMR = 0.06, GFI = 0.92, IFI = 0.97, CFI = 0.97, TLI = 0.96, all greater than 0.9, RMSEA = 0.05, less than 0.08, and the model reflects the relationship between latent variables well. Overall, the empirical results support the research model proposed in this paper, and the conceptual model estimation results are presented in Table 4 and Figure 2.

**TABLE 4:** Summary of path analysis.

Relationships	Unstandardized coefficients	Standardized coefficients	*P*-value	Inferences
Cost → perceived value	0.40	0.44	***	Accepted
Group → perceived value	0.09	0.11	0.02	Accepted
Comment → perceived value	0.16	0.17	***	Accepted
Risk → perceived value	0.24	0.27	***	Accepted
Cost → intention	0.26	0.26	***	Accepted
Group → intention	−0.01	−0.01	0.83	Rejected
Comment → intention	0.18	0.18	***	Accepted
Risk → intention	−0.07	−0.08	0.05	Rejected
Perceived value → Intention	0.64	0.59	***	Accepted

***P < 0.001.

#### Mediating effect of perceived value

In this paper, the mediating effects of perceived value in the hypothesized model were tested for significance using the boot-strapping approach (5,000 replications). The boot-strapping procedure enhances the statistical power of mediation analysis, especially for a small or moderate sample size (Preacher and Hayes, 2008). As presented in Table 5, the direct effects of single-channel shopping cost on perceived value, reference groups on perceived value, positive online reviews on perceived value, single-channel perceived risk on perceived value, and perceived value on purchase intention were all significant. The direct effects of single-channel shopping cost on perceived purchase, positive online reviews on perceived purchase, and perceived value on purchase intention were all significant. Last, the direct relationship between single-channel shopping cost and purchase intention (β = 0.32) is significant when we added perceived value as a mediator. Last, the direct relationship between positive online reviews and purchase intention (β = 0.29) is significant when we added perceived value as a mediator. From the preceding results, we can deduce that perceived shopping partially mediates the relationship between single-channel shopping cost, positive online reviews and purchase intention.

**TABLE 5:** Structural model assessment.

	Estimated effect (SE)				*T*-value	*P*	Test result
Direct effects							
Single-channel shopping cost →perceived value	0.33 (0.03)				10.05***	0.000	Supported
Reference groups →perceived value	0.22 (0.03)				6.54***	0.000	Supported
Positive online reviews →perceived value	0.28 (0.03)				17.51***	0.000	Supported
Single-channel perceived risk → perceived value	0.04 (0.04)				1.10	0.27	Rejected
Perceived value → purchase intention	0.54 (0.03)				15.89***	0.000	Supported
Indirect effects							95% CI^a^
Single-channel shopping cost→ perceived value → purchase intention	0.32 (0.03)						[0.26, 0.39]
Reference groups→ perceived value → purchase intention	0.36(0.03)						[0.29, 0.42]
Positive online reviews→ perceived value→ purchase intention	0.29 (0.03)						[0.23, 0.35]
Model fit	CMIN/DF	RMSEA	RMR	GFI	IFI	CFI	TLI
	2.42	0.05	0.06	0.92	0.97	0.97	0.96

*N* = 620. ***p < 0.001. CI^a^, confidence interval (5,000 bootstrap samples).

#### Moderating effect of contextual factors

To analyze the moderating effect of contextual factors, this paper constructs a moderating effect analysis model. The paper adopts model 14 of SPSS PROCESS v3.3 to analyze the mediating effect of contextual factors. The sample size is set to 5,000, the purchase intention is the dependent variable, the perceived value is the mediator variable, the Contextual factor is the moderator variable, and the purchase cost, reference groups, online comments, and perceived risk are The estimated results of independent variables and moderating effects are shown in Table 6.

**TABLE 6:** Summary of moderated mediation analysis.

Independent variable		Effect	SE	LLCI	ULCI
Single-channel shopping cost	Direct effect	0.1464	0.0295	0.0886	0.2043
	Index of moderated mediation	−0.0244	0.0104	−0.0442	−0.0031
Reference groups	Direct effect	0.0676	0.0288	0.0110	0.1241
	Index of moderated mediation	−0.0223	0.0102	−0.0416	−0.0021
Positive online reviews	Direct effect	0.1408	0.0255	0.0907	0.1908
	Index of moderated mediation	−0.0165	0.0088	−0.0344	−0.0001
Single-channel perceived risk	Direct effect	−0.0008	0.0267	−0.0532	0.0515
	Index of moderated mediation	−0.0229	0.0107	−0.0438	−0.0013

Low situation group = Mean−1 SD, High situation group = Mean + 1 SD.

The results show that contextual factors negatively moderate the mediation effect of single-channel shopping cost on purchase intention via perceived value [index of moderated mediation = −0.0244, SE = 0.0104, 95% CI = (−0.0442, −0.031); direct effect = 0.1464, SE = 0.0295, 95% CI = (0.0886, 0.2043)]. Contextual factors negatively moderate the mediation effect of reference groups on purchase intention via perceived value [index of moderated mediation = −0.0223, SE = 0.0102, 90% CI = (−0.0398, −0.0050); direct effect = 0.676, SE = 0.0288, 95% CI = (0.0200, 0.1150)]. Contextual factors negatively moderate the mediation effect of positive online reviews on purchase intention via perceived value [index of moderated mediation = −0.0165, SE = 0.0088, 90% CI = (−0.0344, −0.0001); direct effect = 0.1408, SE = 0.0255, 95% CI = (0.0907, 0.1908)]. Contextual factors negatively moderate the mediation effect of single-channel perceived risk on purchase intention via perceived value [index of moderated mediation = −0.0229, SE = 0.0107, 90% CI = (−0.0438, −0.0013); direct effect = −0.0008, SE = 0.0267, 95% CI = (−0.0532, 0.0515)].

## Discussion and implications

### Discussion of findings

This study yielded meaningful conclusions. As the results show, single-channel shopping costs have a significant positive effect on the perceived value of purchasing agricultural products omni-channel (β = 0.4, *p* < 0.001). Single-channel shopping cost has a significant positive effect on the purchase intention of purchasing agricultural products omni-channel (β = 0.26, *p* < 0.001). The results are consistent with previous research findings (Guo et al., 2022), indicating that shopping cost is the key factor of perceived value and purchase intention. reference groups has a positive impact on the perceived value (β = 0.09, *p* < 0.05). Positive online reviews have a positive impact on perceived value (β = 0.16, *p* < 0.001). Positive online reviews have a positive impact on perceived intention (β = 0.18, *p* < 0.001). The results show that compared with reference groups, positive online reviews play a more important role in consumers’ purchasing decision processes omni-channel. Single-channel perceived risk has a significant positive effect on the perceived value of purchasing agricultural products omni-channel (β = 0.24, *p* < 0.001).

Second, the study indicates the mediating effect of perceived value on purchase intention. In the model of “single-channel shopping cost→ perceived valueg willingness to buy agricultural products through dual-channel,” the mediating role of perceived value is significant, and the effect size is 0.3219. In the model of “reference groups→ perceived valueo willingness to buy agricultural products omni-channel,” perceived value played a significant mediating role, and the effect size was 0.3555. In the model of “positive online reviews→ perceived valueo willingness to buy agricultural products omni-channel,” perceived value played a significant mediating role, and the effect size was 0.2895. In the model of “single-channel perceived riskg perceived valuee willingness to buy agricultural products through dual-channel,” perceived value played a significant mediating role, and the effect size was 0.4210.

Finally, our results indicate that contextual factors negatively moderate the mediation effect of single-channel shopping cost on purchase intention via perceived value [index of moderated mediation = −0.0244, SE = 0.0104, 95% CI = (−0.0442, −0.031]; direct effect = 0.1464, SE = 0.0295, 95% CI = (0.0886, 0.2043)]. Contextual factors negatively moderate the mediation effect of reference groups on purchase intention via perceived value (index of moderated mediation = −0.0223, SE = 0.0102, 90% CI = (−0.0398, −0.0050); direct effect = 0.676, SE = 0.0288, 95% CI = (0.0200, 0.1150)]. Contextual factors negatively moderate the mediation effect of positive online reviews on purchase intention via perceived value [index of moderated mediation = −0.0165, SE = 0.0088, 90% CI = (−0.0344, −0.0001); direct effect = 0.1408, SE = 0.0255, 95% CI = (0.0907, 0.1908)]. Contextual factors negatively moderate the mediation effect of single-channel perceived risk on purchase intention via perceived value [index of moderated mediation = −0.0229, SE = 0.0107, 90% CI = (−0.0438, −0.0013); direct effect = −0.0008, SE = 0.0267, 95% CI = (−0.0532, 0.0515)].

### Theoretical implications

Recent researchers have begun to emphasize the importance of understanding omni-channel development from a consumer perspective (Hossain et al., 2019; Shi et al., 2020; Guo et al., 2021). This study offers three important theoretical implications for fresh produce omni-channel technology. First, previous research has focused on studies concerning the drivers of search and purchase channels (Arora et al., 2017), rather than the costs and risks of the channel switching process. By combining the research frameworks of VAM and UTAUT, this study fully captures the key factors influencing consumers’ omni-channel purchase intentions. At the same time, it enriches theoretical insights on how consumers’ perceived value affects purchase intention.

More importantly, our study provides new insights into consumer perceived value in a contextually relevant paradigm. The findings confirm the role of positive online reviews and reference groups. Third, while many fresh agri-produce retailers have adopted an omni-channel retail strategy, it is unknown whether the strategy will help retailers drive a positive consumer response (Yan et al., 2020). Focusing on the lesser-focused fresh produce industry, this study seeks to provide theoretical support for these retailers’ strategies.

### Managerial implications

This study may provide several implications for practice. First, agricultural retailers should pay full attention to the role of single-channel shopping costs, which have the greatest impact on consumers’ willingness to purchase agricultural products omni-channel. On the one hand, agriculture retailers can continue to strengthen the construction of online websites to reduce the difficulty of consumers judging product information before purchasing. On the other hand, agricultural retailers should innovate planting technology, improve the quality of agricultural products, continuously reduce the purchase cost of consumers’ dual-channel purchase of agricultural products, and make full use of the impact of purchase costs to increase consumers’ willingness to purchase.

Second, agricultural retailers should pay more attention to social influence. Agricultural retailers should choose high-quality websites and anchors with good reputations to carry out online sales. High-quality online reviews should be selected, influential positive online reviewers should be actively discovered, and positive information about product differences and user experience should be promptly delivered to potential consumers. Agriculture retailers can also take corresponding selected comment incentive measures to encourage consumers to post positive online reviews, improve the quantity and quality of positive online reviews on agricultural products, further establish smooth communication channels among customers, and make full use of social influence.

Third, agriculture retailers should keep abreast of consumers’ single-channel perceived risk. Enterprises should pay attention to the investigation of the consumer market and strive to accurately grasp the source of consumers’ single-channel perceived risk. In addition, retailers should ensure consistency of information across channels to reduce uncertainty in omni-channel technology and shopping processes. For example, establish cross-channel feedback mechanisms to meet customer needs and improve competitive advantage.

Finally, the perceived value of consumers should be continuously improved. The process of consumer selection is essentially a process of seeking value. Retailers should enhance their fresh produce logistics and distribution systems to avoid problems such as untimely deliveries and product spoilage. Consumers can reap the same fresh produce whether in-store or at home, thus facilitating a seamless and consistent shopping experience.

### Limitations and future research

Although the results of this study have provided some implications for agricultural product dual-channel development in China, there are still some limitations. Future research could take the following aspects into account.

Most of the research hypotheses and conceptual models in this paper are based on the general online shopping theory and are not very targeted. Future research could consider further modifying and improving the model from the characteristics of omni-channel shopping agricultural products and the characteristics of omni-channel consumers to improve the pertinence and explanatory power of the model in the agricultural product sales model.

In studying the factors of consumers’ channel choice, we compared the attributes of a single channel and omni-channel. This study focuses on the factors that have a significant effect on perceived value. Future research will focus on some other factors that affect consumer attitudes and perceptions of consumers’ willingness to choose channels. such as agricultural product types and green agricultural products.

## Data availability statement

The raw data supporting the conclusions of this article will be made available by the authors, without undue reservation.

## Author contributions

YL designed the study, drafted the initial manuscript, and contributed to the revised manuscript. SZ collected the data, performed statistical analysis, and drafted the initial manuscript. Both authors discussed the results and contributed to the final manuscript.

## Footnotes

1 https://www.cfsn.cn/front/web/site.searchshow?pdid=154&id=60047 (accessed 9 Aug, 2021)

2 https//www.100ec.cn/zt/2021zgsxdsscsjbg/ (accessed 3 Mar, 2022)

## Appendix

### Questionnaire items

**Table:**

Construct	Item	Source
Single-channel shopping cost	SSC1: Buying agricultural products omni-channel, the price is more transparent.	Wood and Scheer, 1996; Jepsen, 2007
	SSC2: It is more convenient to compare and evaluate agricultural products, when purchase omni-channel.	
	SSC3: It takes less time and effort to collect information, when purchase omni-channel.	
	SSC4: It is less likely to perform poorly in after-sales service and quality inspection, when purchase omni-channel.	
Reference groups	RG1: I will listen to the opinions of those around me.	Bearden and Etzel, 1982; Escalas and Bettman, 2005
	RG2: I will listen to the opinions of my family and friends.	
	RG3: I will follow the recommendation of the live-streaming broadcaster.	
	RG4: I will choose the one with more purchases.	
Positive online reviews	POR1: I especially value positive online reviews.	Hennig-Thurau et al., 2010; Fan et al., 2020
	POR2: I especially value the number of positive reviews.	
	POR3: I especially value the level of detail in positive reviews.	
	POR4: I particularly value product quality positive description reviews.	
	POR5: I particularly value positive reviews of the consumer experience.	
Single-channel perceived risk	SCP1: Less likely to buy fake and shoddy agricultural products omni-channel.	Stone and Grønhaug, 1993; Corbitt et al., 2003
	SCP2: Less likely to buy low-quality agricultural products omni-channel.	
	SCP3: Less likely to buy defective agricultural products omni-channel.	
	SCP4: The price of agricultural products purchased omni-channel is lower.	
	SCP5: Vendors are less likely to launch more exciting products soon.	
Perceived value	PV1: It is more efficient to purchase agricultural products omni-channel	
	PV2: The quality of agricultural products purchased omni-channel is guaranteed.	Davis et al., 1992; Voss et al., 1998; Neal, 1999
	PV3: Buying agricultural products omni-channel saves money.	
	PV4: Buying agricultural products omni-channel is fun.	
	PV5: Buying agricultural products omni-channel earns me more recognition and compliments.	
Purchase intention	PI1: I consider purchase agricultural products omni-channel.	Cronin et al., 2000
	PI2: I would like to recommend omni-channel buying to friends and family.	
	PI3: I think it is very convenient to buy agricultural products omni-channel.	
Contextual factors	CF1: The cost of purchasing agricultural products online has not increased significantly.	Dhar, 1992; Zepeda and Deal, 2010
	CF2: I am fully qualified to buy agricultural products through the Internet.	
	CF3: The stronger the government’s supervision, the more willing to buy agricultural products online.	
	CF4: I think it is very convenient to buy agricultural products online.	
